# Development of a program theory for shared decision-making: a realist review protocol

**DOI:** 10.1186/s13643-017-0508-5

**Published:** 2017-06-17

**Authors:** Gary Groot, Tamara Waldron, Tracey Carr, Linda McMullen, Lori-Ann Bandura, Shelley-May Neufeld, Vicky Duncan

**Affiliations:** 1 Department of Community Health and Epidemiology, Health Sciences Building, University of Saskatchewan College of Medicine, 107 Wiggins Road, Saskatoon, SK S7N 5E5 Canada; 2 Department of Psychology, College of Arts and Sciences, University of Saskatchewan Arts 154, 9 Campus Drive, Saskatoon, SK S7N 5A5 Canada; 3 Patient Consultant, Loreburn, Canada

**Keywords:** Shared decision-making, Realist review, Mechanisms, Quality improvement, Health systems, Medical decision-making

## Abstract

**Background:**

The practicality of applying evidence to healthcare systems with the aim of implementing change is an ongoing challenge for practitioners, policy makers, and academics. Shared decision- making (SDM), a method of medical decision-making that allows a balanced relationship between patients, physicians, and other key players in the medical decision process, is purported to improve patient and system outcomes. Despite the oft-mentioned benefits, there are gaps in the current literature between theory and implementation that would benefit from a realist approach given the value of this methodology to analyze complex interventions. In this protocol, we outline a study that will explore: “In which situations, how, why, and for whom does SDM between patients and health care providers contribute to improved decision making?”

**Methods:**

A seven step iterative process will be described including preliminary theory development, establishment of a search strategy, selection and appraisal of literature, data extraction, analysis and synthesis of extracted results from literature, and formation of a revised program theory with the input of patients, physicians, nurse navigators, and policy makers from a stakeholder session.

**Discussion:**

The goal of the realist review will be to identify and refine a program theory for SDM through the identification of mechanisms which shape the characteristics of when, how, and why SDM will, and will not, work.

**Systematic review registration:**

PROSPERO CRD42017062609

**Electronic supplementary material:**

The online version of this article (doi:10.1186/s13643-017-0508-5) contains supplementary material, which is available to authorized users.

## Background

Shared decision-making (SDM) is a model of decision-making that focuses on a balanced relationship between patients and physicians in order to make a mutually agreed upon medical treatment decision [1, 2]. SDM emerged in the 1980s and 1990s largely in reaction to the paternalistic model that dominated at the time [1]. While there is considerable variation in SDM definitions [1, 3–5], there are recognized common themes, including patient autonomy [1, 6], dynamic/interactive exchange between the patient and physicians [7, 8], discussion of preferences and treatment options [1, 2, 9, 10], and mutual consensus on treatment decisions [2, 11].

SDM effectiveness has been extensively assessed; however, there is variable evidence that SDM improves the quality of care for a given patient [12, 13]. Durand and colleagues specifically assessed the ability for SDM to increase an array of patient outcomes, including knowledge and participation, for socioeconomically disadvantaged groups compared to higher status patients [14]. The authors also noted that all patients had an increase in decision quality, reflected by reduced decisional conflict [14]. A separate systematic review found that SDM was more effective when applied to patients with complex and chronic illnesses, such as cancer [15]. The variability in these findings support the notion that the context of implementation is critically important for the successful use of SDM.

A particularly well-cited model of SDM, the interprofessional shared decision-making model (IP-SDM) [16–18], focuses on all individuals who may be involved in the decision making-process including those outside of the patient–physician dyad, such as family, friends, and other health providers. Developed to address the shortcomings of previous frameworks, IP-SDM incorporates interprofessionality and the involvement of patient supports [19]. This reflects the fact that decision-making is very context-dependent by considering environmental influences (i.e., social norms, organizational routines, and institutional structures) on decision-making [16]. While not explicitly stated, the key presumption behind IP-SDM that is collaboration between health care provider(s) and patients in order to exchange knowledge and preferences will result in the formation of an ideal patient-centered decision in a given situation [16].

Despite extensive research in the field of SDM, there is little systematic integration of SDM into clinical practice [20–22]. Evidence varies in both consistency of how SDM is defined [1–3] and implemented [23–26]. A recent study concluded that implementation of IP-SDM has struggled as the framework, and users of the framework, lack conceptual clarity and minimal implementation guidance [19]. A study conducted by Lloyd and colleagues in 2013 demonstrated the difficulty in SDM implementation, as SDM is a very complex intervention and pre-existing contexts can drastically change implementation success [27]. Further, the authors discussed how many implementers confuse patient decision supports, such as decision aids, as SDM implementation. While SDM may include decision aids, it is important to fully understand the implication of successful, and unsuccessful, delivery of such complex programs. The need for this review was also prompted by the policy decision of the provincial government’s Appropriateness of Care committee to adopt SDM system wide. The lead author, as the clinical co-lead of this committee, advocated for the importance of a theory-based policy and has engaged our team to conduct this review. To begin this, we conducted a scoping review [28] of literature [29], where we identified a gap between theoretical models, such as IP-SDM, and empirical evidence regarding how models work, for whom and in what circumstances.

As a consequence of the current state of literature, there is a lack of testable hypotheses of how, why, and in which contexts SDM is successful. To bridge this gap, we intend to conduct a realist review that draws on both primary and secondary data sources. Embedded in realist philosophy is the acknowledgement that the world is “real” but is perceived, interpreted, and responded to through human processes such as language and culture. Realist methodology accounts for both “reality” and human responses to reality in how outcomes are understood [30]. Originally developed by Pawson to systematically explore how contextual factors influence the link between an intervention and outcomes, realist reviews answer the question “what works, how, for whom, in what circumstances, and to what extent?” [31–33]. This is achieved by the output of a program theory, a model which describes and explains the nuances of the specific process of interest. The identification and refinement of a program theory forms clear and testable hypotheses that can be used to inform policy makers and to assist researchers in the design of subsequent robust studies.

The use of realist review makes program theory explicit by developing testable hypotheses about how, for whom, and in which contexts a given intervention or program is thought to work [31–33]. Realist reviews extend past the traditional boundaries enforced in systematic review by incorporating multiple forms of sources, instead of limiting to peer-reviewed sources only. Further, and potentially most noteworthy, realist reviews move past simply describing literature and aims to unpack how complex programs are successful, or unsuccessful, and the contexts which impact their success [34]. This makes realist reviews specifically appropriate for SDM which is a complex intervention with success highly dependent on implementation.

The proposed realist review will allow the formation of testable hypotheses to assist in forming a stronger understanding of SDM. The results of the review will identify hypotheses for subsequent realist evaluations using the following configuration: a context (C) that triggers a mechanism (M) leading to an outcome (O) [31–33]. Contexts (specific circumstances and factors that are pre-existing to a condition) interact with mechanisms (processes, often psychological, that are triggered) to cause outcomes (the results of the interaction between the present contexts and mechanisms). Understanding that mechanisms underlying causality are context-dependent is a crucial component of realism [31, 35]. Using a CMO configuration, researchers are able to gain understanding of the successes and failures of an intervention, and the vital interplay between contexts, mechanisms, and outcomes.

In realist research, CMO configurations represent a type of middle-range theory. Middle-range theory is a level of theory abstraction that describes uniformities of social behavior that can be expanded to form testable hypotheses by configuring features of an intervention together [31]. Middle-range theories are those abstracted only to the point that they are still able to be observed and may still be incorporated into propositions [36]. Incorporating CMO configurations as middle-range theories generates causative explanations of outcomes in a program theory [37], which can be tested to confirm, refute, or refine hypotheses. Our research team believes that middle-range theories will exist within current literature in non-CMO configurations. Identifying and building on CMO configurations as middle-range theories [36] will be critical for broader and effective SDM implementation. CMO configurations, therefore, construct the foundation of realist reviews [36, 37].

Our team will use realist review methodology to conduct a theory-driven knowledge synthesis. This will enable the development of a program theory that is testable at the intervention level and provide the foundation for testable hypotheses applicable across a range of health care contexts. For example, testable hypotheses will be derived for different clinical subgroups, such as oncology and mental illness. Oncology offers a good example of complex decision-making given the nature of the disease and the multiple, time-constrained decisions that need to be made [38, 39], heightening the reliance on patient values and preferences. We will therefore examine oncology literature to build an understanding of SDM in complex situations. However, our program theory will be developed based on SDM literature from all potential fields. This will in turn establish a program theory of SDM illuminating how improved decision-making can be achieved.

As realist review methodology is complex and still evolving, this protocol outlines a detailed process to add to the evolution of this research methodology. Depicting the process will ensure that this review follows a literature informed and consistent methodology. The purpose of the study described in this protocol is to develop a program theory of SDM using realist review methodology. Throughout this process, we intend to iteratively focus our review, guided by any middle-range theories that exist in the SDM literature that we will reformulate into CMO configurations. By employing realist methodology, we will address the following question: “In which situations, how, why, and for whom does SDM between patients and health care providers contribute to improved engagement in the shared decision - making process?” Improved engagement in the shared decision-making process will be specifically related to having a decision that is patient-centered and patient-informed. To address these issues, we will explore:

1. What mechanisms facilitate or hinder engagement in the SDM process?
2. What contexts impact the expression of the identified mechanisms?
3. When do contexts and mechanisms form specific outcomes?

## Methods

**Fig. 1:**
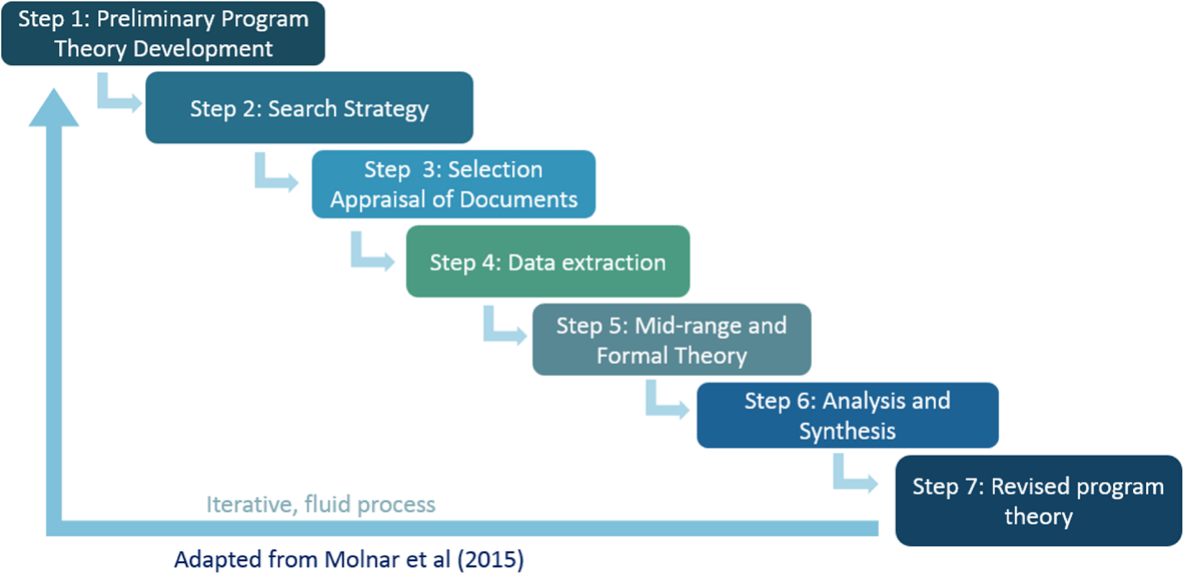
Realist review process. Adapted from Molnar and colleagues [40], this depicts the seven steps undertaken to formulate a shared decision-making framework from initial search strategy development through stakeholder testing and refinement

As a team of clinicians, content specialists, a health science librarian, and other researchers, we will approach SDM literature with the intention of developing a program theory. Adapting Molnar’s process for realist reviews [37, 40], we will (1) develop a preliminary program theory, (2) develop a search strategy, (3) select and appraise literature in accordance with realist methodology [37, 41], (4) extract data, (5) identify relevant middle-range and formal theories, (6) perform data analysis and synthesis, and (7) form a revised program theory with the input of stakeholders (see Fig. 1). Following RAMESES quality standard guidelines for an “excellent” program theory [42, 43], we will identify the relationship linking the program theory with formal theory (existing theories used to understand interventions), describe the implications of the revised program theory, and develop a revised program theory comprised of multiple CMO configurations. The completion of this seven-step process will create a program theory supported by the current literature and the perspectives of knowledge users (such as patients, policy makers, and health care providers). A PRISMA-P checklist is attached as an additional file (see Additional file 1).

### Preliminary program theory development

The preliminary program theory will be derived from the preliminary results of our completed scoping review [29]. Our research team will outline the process and outcomes of SDM according to empirical and theoretical accounts of patient and provider experiences. This will be completed by utilizing preliminary information gathered from the purposive scope of the literature, along with team collaboration depicting the hypotheses of how, when, and for whom SDM may be successfully implemented. At this stage, we will consult with realist methodologist, Dr. Gill Westhorp, to guide and inform further theory development through the identification of the key program components. Content experts will be consulted once the program theory has been developed, as described below.

### Search strategy development

**Fig. 2:**
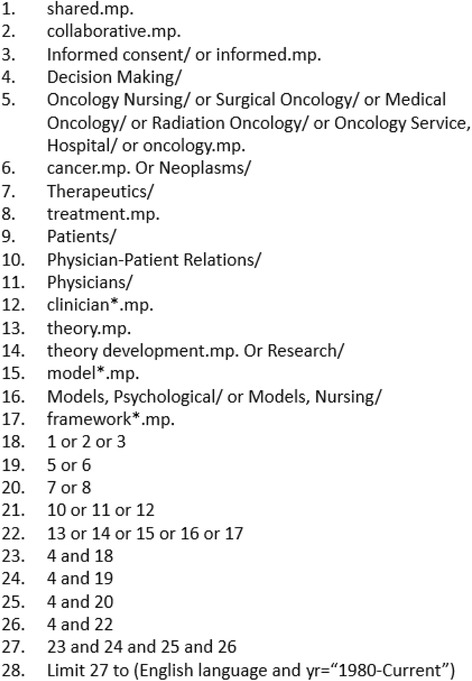
Medline search strategy. Search strategy conducted for scoping review using Medline

Our next step will be a purposive examination of the SDM literature, performed by two team members, beginning with a search using Medline and Google Scholar as it is believed this will provide the most relevant documents. This search is purposive as it is targeting articles focused on shared decision-making and looking for those that provide the most valuable information. Search terms will include: “shared,” “collaborative,” “decision making,” “informed,” “oncology,” “cancer,” “treatment,” “patient(s),” “physician(s),” “clinician(s),” “theory,” “development,” “model(s),” and “framework(s).” The literature on decision-making in cancer will be targeted as an example of complex decision-making, where short timeframes and urgent circumstances exist. However, literature outside of cancer care will also be considered. These search terms were identified by the authors during the scoping review. This preliminary search strategy will be refined as necessary, at the discretion of the two researchers in relation to the relevance of articles returning. An example of the search strategy for Medline can be found in Fig. 2 (see Fig. 2). All identified sources will have references stored within EndNote.

Using snowball sampling, our team will perform reference searches from the key articles, determined by articles highly cited throughout the SDM field. We will also examine additional publications by influential authors determined by amount of citations, such as France Légaré and Dawn Stacey. Following realist review methodology, that acknowledges non-academic sources as valid, our team will additionally search the gray literature. The use of gray literature often delves into the innermost processes of interventions in greater, more pragmatic detail [32], especially in areas hard to access through traditional searches of published literature. We will search the gray literature from North American health jurisdictions. Exact search criteria for gray literature will be developed upon completion of the initial search.

Once we have performed our initial search, we will determine if a secondary search will be performed. The research team will determine necessity based on the depth and extensiveness of literature collected. If the research team members conclude further searching is necessary, two team members, along with the librarian, will expand search terms and utilize other databases, such as EMBASE.

### Literature selection and appraisal

To reduce selection bias, two members of our team will independently screen reference and information resources using the following inclusion criteria: (1) exchange between a patient and/or patient’s family and a health care provider, (2) a clinical situation where the patient is legally competent to make their own decision, (3) adult patients (18 years or older) making decisions about their own medical situation, (4) sources from 1980 to present, (5) English-language sources only (due to the language constraints of our team). We will exclude studies on that focus on surrogate decision-making, when patients are unable to be involved (e.g., end-of-life care, pediatric decision-making, reduced competency, and dementia). Reviewers will also use Pawson’s criteria of relevance (the applicability of the study to the theory in question) and rigor (methodological appropriateness in relation to the credibility and trustworthiness of approaches) to assess each source [43]. Rigor will be assessed by determining if the document was conducted in a methodologically sound approach, in relation to what the document’s authors designed.

Screening will be iterative, beginning with all abstracts followed by gray literature and full-text review of identified abstracts. When two members cannot agree on the inclusion of a source, a third member will assess the source to recommend its inclusion or exclusion. Any additional sources that arise from potential additional searches will be reviewed using the same strategy.

### Data extraction

To extract data from the sources, two research team members will independently read each source in full to identify the primary and relevant outcomes from each source. Explanatory accounts (EA) will be extracted in the form of “if-then” (phrased as if “*x*” occurs, then “*y*” will be the result) propositions [44, 45]. EA statements will be selected as those findings that authors noted as a primary outcome or important outcome in relation to impact on SDM. Findings that are not able to be translated into an EA statement will be extracted in verbatim format and included as such. Researchers will follow an extraction template that will include (1) article bibliographic information, (2) relevant study/document notes (its relevance to the program theory), (3) country of study/document, (4) focus/sample of study/document (including if the focus is on patients, health care provider, both, or systematic factors), (5) if a research article or if the study was empirical or theoretical, and (6) all EA statements extracted from each paper. All extraction records will be managed using Microsoft Excel. Authors will pilot this extraction template by independently extracting information from approximately ten articles, then discussing the results. Refinement of the extraction template may occur following piloting if necessary.

### Identification of middle-range and formal theories

We will identify middle-range and formal theories from the literature review by noting those used within any source. Existing middle-range theories will be reformed into CMO configurations to build a stronger program theory. Where possible, this will be supported by formal theory [43], such as cognitive and behavioral theories, to understand the causal relationship between mechanisms and outcomes. If we are unable to determine formal theories at the outset, our team will find such theories once a revised program theory is developed, as recommended by the realist review guidelines [37]. This will be completed by performing additional searches, as required, within medical decision-making and psychological theory, utilizing the researcher team’s expertise (Drs. Carr and McMullen). Through this process, we will develop middle-range theories with testable hypotheses, supported by formal theory.

### Analysis and synthesis process

Pattern identification is important in realist reviews [40] as it allows researchers to recognize key mechanisms that may impact the success or failure of a specific intervention’s implementation. Extracted EA statements will be examined by two researchers to identify recurrent themes within mechanisms and grouped to ascertain emerging patterns of CMOs. Examples of thematic groups might be “patient anxiety” or “health care providers’ perception of time”. EA statements that fit into more than one thematic group will be included in all relevant groups. For example, if an EA statement had themes of “patient anxiety” and “patient education level,” the statement would appear in both thematic groups to ensure that EA statements are reviewed for patterns within all appropriate thematic contexts.

Following extraction and consolidation of our findings into themed EA statements, our research team will synthesize the data into CMO configurations [32]. As EA statements will vary depending on the detail provided in each source, CMOs are likely to reside in the “*x*/if” and/or “*y*/then” side of the “if *x*–then *y*” configuration explained above. However, it is likely that O will be heavily based within the “*y*” domain, while “*x*” will describe C and M (hidden and explicitly stated). Hidden mechanisms will be inferred from the content and clinical expertise within our team. Our team will isolate identified mechanisms and determine how they interact within the procedural steps of IP-SDM, outlined by Légaré [16]. This will determine the SDM mechanisms variably affecting outcomes within particular contexts for different individuals and potentially how interprofessional involvement impacts the process. The intent of this stage determines the major contributing CMOs to successful, or unsuccessful, implementation of SDM.

CMO configurations will collectively shape our program theory. Guided by literature, our research team of content specialists will then identify which mechanisms are thought to be the “key” in the SDM process. These key mechanisms, anticipated to be processes such as patient anxiety, will be those believed to have a strong impact, either positively and negatively, on the implementation of SDM within medical decision-making. These key mechanisms will be used to revise our preliminary program theory, which will explain how varying contexts may change how these key mechanisms facilitate, or hinder, gradient outcomes (that is facilitate/hinder at varying strengths).

### Stakeholder input and dissemination of revised program theory

Once our revised program theory has been developed, our team will first consult experts within the fields of SDM and realist research, Dr. France Légaré, Dr. Gill Westhorp. Dr. Légaré is currently the Canadian Chair of shared decision-making, and the research team has made contact with her and her team in this regard. We will present the program theory to both expert groups in separate consultations to gather their insights on SDM and realist review. Content experts will be asked if the program theory reflects their understanding of the SDM literature. The methodology expert will ensure that the program theory meets realist review standards. We will document these consultations and use the data from these experts to further confirm, refine, or refute our program theory.

We will engage stakeholders who are involved in medical decisions to offer feedback on our program theory. These stakeholders will be physicians, nurse navigators, patients, and policy makers, recruited through the local health region and provincial health ministry. The stakeholder session will be conducted on a single day. All individuals will participate in a single group to further understand the interplay that exists between parties, and how this may impact the identified CMO configurations. Combining participants from an assortment of backgrounds will provide the opportunity to hear from each group that is most directly impact by SDM and afford the participants the opportunity to hear, and react to, the perspectives of others in the SDM process. This session will be semi-structured and audio-recorded. By hosting a stakeholder session with physicians, nurse navigators, patients, and policy makers, we will follow a model developed by Harris and colleagues [46] for participatory realist synthesis. We intend to seek stakeholder perspectives on the mechanisms identified through our literature search, as well as their views on how the CMO configurations within our program theory reflect their experiences. Specifically, we seek to gain their opinion on whether the most relevant mechanisms have been identified, if the mechanisms are depicted in a contextually appropriate manner, and if there are CMOs that we have overlooked. Engaging stakeholders in a realist review is shown to improve findings by identifying active components and areas to tailor in the intervention, describing feedback loops which influence intervention success, and analyzing ways in which contexts affect the intervention [46]. Our research team will use the data from the stakeholder session to confirm, refute, or refine our program theory.

## Discussion

This protocol outlines the methodological steps to complete a realist review assessing “In which situations, how, why, and for whom does SDM between patients and health care providers contribute to improved decision making?” The focus of this research is to develop a program theory encompassing the CMOs of when, how, and why SDM is successfully implemented. Using CMO configurations, we will build on IP-SDM to create a more robust understanding of SDM. We will be complying with RAMESES methodological guidelines for realist reviews to produce a sound framework of SDM. Through the identification of mechanisms underlying SDM, implementation of SDM will be more clearly defined, increasing accessibility for knowledge users, such as patients, health care providers and policy makers.

Forming a realist-based SDM program theory will contribute to current scholarly knowledge in medical decision-making. Identifying contexts, mechanisms, and outcomes through current literature and key stakeholders, our team will expand the current knowledge of SDM launching further research into SDM implementation within health systems. Study results will be disseminated through peer-reviewed journals, conferences, and as part of a graduate thesis. This research may enhance the uptake and impact of SDM use in medical decision-making. It is anticipated that continued research in this area will improve quality of care for patients and increase efficiency within health care systems.

Once the SDM program theory has been developed, the authors intend to conduct realist evaluations in a number of contexts. We plan to examine how the program theory aligns within both fields of mental illness and indigenous health. Further, the program theory will be tested within local cancer centers, to confirm, refine, or refute CMO configurations. It is anticipated that the program theory will continually be revised to identify relevant contexts that impact mechanisms in varying manners.

## Additional file

Additional file 1: PRISMA-P checklist. PRISMA-P 2015 Checklist: recommendations for prospective authors of systematic reviews. (DOCX 29 kb)

## Abbreviations

CMO: Context-mechanism-outcome
EA: Explanatory account
IP-SDM: Interprofessional shared decision-making
SDM: Shared decision-making
SHR: Saskatoon Health Region

## References

[1] Charles C, Gafni A, Whelan T (1997). Shared decision-making in the medical encounter: what does it mean? (or it takes at least two to tango). Soc Sci Med.

[2] Elit L, Charles C, Gold I, Gafni A, Farrell S, Tedford S (2003). Women’s perceptions about treatment decision making for ovarian cancer. Gynecol Oncol.

[3] Stacey D, Pomey MP, O'Connor AM, Graham ID (2006). Adoption and sustainability of decision support for patients facing health decisions: an implementation case study in nursing. Implement Sci.

[4] Menard C, Merckaert I, Razavi D, Libert Y (2012). Decision-making in oncology: a selected literature review and some recommendations for the future. Curr Opin Oncol.

[5] Stacey D, Samant R, Bennett C (2008). Decision making in oncology: a review of patient decision aids to support patient participation. CA Cancer J Clin.

[6] King JS, Moulton BW (2006). Rethinking informed consent: the case for shared medical decision-making. Am J Law Med.

[7] Nannenga MR, Montori VM, Weymiller AJ, Smith SA, Christianson TJ, Bryant SC (2009). A treatment decision aid may increase patient trust in the diabetes specialist. The Statin Choice randomized trial. Health Expect.

[8] Charles C, Gafni A, Whelan T (1999). Decision-making in the physician-patient encounter: revisiting the shared treatment decision-making model. Soc Sci Med.

[9] King JS, Eckman MH, Moulton BW (2011). The potential of shared decision making to reduce health disparities. J Law Med Ethics.

[10] Coulter A (1999). Paternalism or partnership? Patients have grown up—and there’s no going back. BMJ.

[11] Frosch DL, Kaplan RM (1999). Shared decision making in clinical medicine: past research and future directions. Am J Prev Med.

[12] Legare F, Moumjid-Ferdjaoui N, Drolet R, Stacey D, Harter M, Bastian H (2013). Core competencies for shared decision making training programs: insights from an international, interdisciplinary working group. J Contin Educ Health Prof.

[13] Kane HL, Halpern MT, Squiers LB, Treiman KA, McCormack LA (2014). Implementing and evaluating shared decision making in oncology practice. CA Cancer J Clin.

[14] Durand MA, Carpenter L, Dolan H, Brave P, Mann M, Bunn F (2014). Do interventions designed to support shared decision-making reduce health inequalities? A systematic review and meta-analysis. Public Library Sci One.

[15] Joosten EAG, DeFuentes-Merillas L, de Weert GH, Sensky T, van der Staak CPF, de Jong CAJ (2008). Systematic review on the effects of shared decision-making on patient satisfaction, treatment adherence and health status. Psychother Psychosom.

[16] Legare F, Stacey D. Interprofessional Shared Decision Making (IP-SDM) Model. The Ottawa Hospital: Research Institute. 2010. https://decisionaid.ohri.ca/ip-sdm.html.

[17] D’Amour D, Ferrada-Videla M, San L, Rodriguez M, Beaulieu MD (2005). The conceptual basis for interprofessional collaboration: core concepts and theoretical frameworks. J Interprof Care.

[18] Légaré F, & Stacey D, Legare F, Stacey D. IP-SDM concepts defined. The Ottawa Hospital: Research Institute. 2010. https://decisionaid.ohri.ca/ip-sdm.html.

[19] Dogba MJ, Menear M, Stacey D, Briere N, Legare F (2017). The evolution of an interprofessional shared decision making research program: reflective case study of an emerging paradigm. J Interprof Care.

[20] Charles C, Gafni A, Whelan T. Self-reported use of shared decision-making among breast cancer specialists and perceived barriers and facilitators to implementing this approach. Health Expectations. 2004;7(4):338–48. 10.1111/j.1369-7625.2004.00299.x PMC5060255 15544686

[21] Elwyn G (2006). Idealistic, impractical, impossible? Shared decision making in the real world. Br J Gen Pract.

[22] Elwyn G, Frosch D, Thomson R, Joseph-Williams N, Lloyd A, Kinnersley P (2012). Shared decision making: a model for clinical practice. J Gen Intern Med.

[23] Levinson W, Kao A, Kuby A, Thisted RA (2005). Not all patients want to participate in decision making. J Gen Intern Med.

[24] Kehl KL, Landrum MB, Arora NK, Ganz PA, van Ryn M, Mack JW (2015). Association of actual and preferred decision roles with patient-reported quality of care: share decision making in cancer care. JAMA Oncology.

[25] Heyland DK, Tranmer J, O’Callaghan CJ, Gafn A (2003). The seriously Ill hospitalized patient: preferred role in end-of-life decision making?. J Crit Care.

[26] Jenkins V, Fallowfield L, Saul J. Information needs of patients with cancer: results from a large study in UK cancer centres. Br J Cancer. 2001;84(1). 10.1054/bjoc.2000.1573 PMC2363610 11139312

[27] Lloyd A, Joseph-Williams N, Edwards A, Rix A, Elwyn G (2013). Patchy ‘coherence’: using normalization process theory to evaluate a multi-faceted shared decision making implementation program (MAGIC). Implement Sci.

[28] Berg RC, Nanavati J (2016). Realist review: current practice and future prospects. J Res Pract.

[29] Waldron T, Groot G. Shared decision making and decision aids. 2015. Unpublished.

[30] Pawson R, Tilley N. Realist evalutation. Community matters: British Cabinet Office; 2004.

[31] Pawson R, Tilley N. Realistic evaluation. Thousand Oaks, California: SAGE Publications Ltd; 1997.

[32] Pawson R (2006). Evidence-based policy: a realist perspective.

[33] Pawson R (2013). The science of evaluation: a realist manifesto.

[34] Pawson R, Greenhalgh T, Harvey G, Walshe K (2005). Realist review––a new method of systematic review designed for complex policy interventions. J Health Serv Res Policy.

[35] Dalkin SM, Greenhalgh J, Jones D, Cunningham B, Lhussier M (2015). What's in a mechanism? Development of a key concept in realist evaluation. Implement Sci.

[36] Merton R (1968). Social theory and social structure.

[37] Jagosh J, Pluye P, Wong G, Queen M, McGill University Participatory Research at McGill, McGill University Participatory Research at McGill (2016). Critical reflections on realist review: insights from customizing the methodology to the needs of participatory research assessment. Res Synth Methods.

[38] Coulter A. Patient information and shared decision-making in cancer care. British Journal of Cancer. 2003;89(Suppl 1):S15–S16. 10.1038/sj.bjc.6601080 PMC2753004 12915899

[39] Reyna VF, Nelson WL, Han PK, Pignone MP. Decision making and cancer. Am Psychol. 2015;70(2). 10.1037/a0036834 PMC4347999 25730718

[40] Molnar A, O’Campo P, Ng E, Mitchell C, Muntaner C, Renahy E (2015). Protocol: realist synthesis of the impact of unemployment policies on poverty and health. Eval Program Plann.

[41] Wong G, Greenhalgh T, Westhorp G, Pawson R. Development of methodological guidance, publication standards and training materials for realist and meta-narrative reviews: the RAMESES (Realist and Meta-narrative Evidence Synthesis - Evolving Standards) project. Health Services and Delivery Research. 2014;2(30). 25642521

[42] Wong G, Greenhalgh T, Westhorp G, Buckingham J, Pawson R (2013). RAMESES publication standards: realist syntheses. BMC Med.

[43] Wong G, Westhorp G, Pawson R, Greenhalgh T. Realist synthesis: RAMESES training materials. RAMESES; 2013. http://www.ramesesproject.org/media/Realist_reviews_training_materials.pdf​.

[44] Pearson M, Brand SL, Quinn C, Shaw J, Maguire M, Michie S (2015). Using realist review to inform intervention development: methodological illustration and conceptual platform for collaborative care in offender mental health. Implement Sci.

[45] Jackson S, Kolla G. A new realistic evaluation analysis method: Linked coding of context, mechanism, and outcome relationships. 2012;33(3): 339–49.

[46] Harris J, Croot L, Thompson J, Springett J. How stakeholder participation can contribute to systematic reviews of complex interventions: figure 1 (PDF download available). 2016. 10.1136/jech-2015-205701 PMC4752615 26475921

